# A Review of Cervidae Visual Ecology

**DOI:** 10.3390/ani14030420

**Published:** 2024-01-27

**Authors:** Blaise A. Newman, Gino J. D’Angelo

**Affiliations:** Warnell School of Forestry and Natural Resources, University of Georgia, Athens, GA 30602, USA

**Keywords:** Cervidae, deer, visual ecology, predator–prey, foraging, movement, communication, human–deer interactions

## Abstract

**Simple Summary:**

The purpose of our review is to bridge the research gaps between cervid physiology and ecology by offering a comprehensive review of cervid visual ecology that emphasizes the interplay between the visual adaptations of cervids and their interactions with habitats and other species. Ultimately, a better understanding of cervid visual ecology allows researchers to gain deeper insights into their behavior and ecology, providing critical information for conservation and management efforts.

**Abstract:**

This review examines the visual systems of cervids in relation to their ability to meet their ecological needs and how their visual systems are specialized for particular tasks. Cervidae encompasses a diverse group of mammals that serve as important ecological drivers within their ecosystems. Despite evidence of highly specialized visual systems, a large portion of cervid research ignores or fails to consider the realities of cervid vision as it relates to their ecology. Failure to account for an animal’s visual ecology during research can lead to unintentional biases and uninformed conclusions regarding the decision making and behaviors for a species or population. Our review addresses core behaviors and their interrelationship with cervid visual characteristics. Historically, the study of cervid visual characteristics has been restricted to specific areas of inquiry such as color vision and contains limited integration into broader ecological and behavioral research. The purpose of our review is to bridge these gaps by offering a comprehensive review of cervid visual ecology that emphasizes the interplay between the visual adaptations of cervids and their interactions with habitats and other species. Ultimately, a better understanding of cervid visual ecology allows researchers to gain deeper insights into their behavior and ecology, providing critical information for conservation and management efforts.

## 1. Introduction

The perceptual space of an animal is determined by the world it perceives through its various sensory systems, including its visual system, which influences its decision-making processes and behaviors [1,2]. Visual perception in animals is attributable to multiple visual aspects such as acuity, contrast sensitivity, color discrimination, and depth perception. Photoreceptors provide the basis for the visual perception of color [3], the discrimination of fine and coarse details [4], and motion detection, while retinal ganglion cells promote the spatial integration of information across the visual field [5]. The ability of animals to perceive and integrate visual information across space (i.e., spatial resolution) and time (i.e., temporal resolution) is fundamental to their ecology and shapes both inter- and intra-specific interactions [6,7]. For example, an animal’s ability to accurately detect and predict the motion of another animal might be crucial in determining the outcome of predator–prey interactions [8,9,10] or in locating conspecifics and mates [11].

The visual information available to an animal is constrained by the physiological properties of its sensory system (e.g., photoreceptor density) and the physical characteristics of its environment (e.g., light environment). For example, the orange-yellow pelage of a tiger (*Panthera tigris*) contrasts sharply against a predominantly green backdrop for a trichromat, while many dichromats would perceive minimal color contrast [12]. The high color contrast trichromats experience in the described scenario is a result of their possession of both M- and L-cone photoreceptors. In general, cone photoreceptors fall within three different spectral types that are maximally sensitive to varying wavelengths of light depending on their exact structure: short-wavelength sensitive S-cones (blue), medium-wavelength sensitive M-cones (green), and long-wavelength sensitive L-cones (red) [7]. Dichromats often have either an M- or L-cone photoreceptor, not both, and perceive less contrast between mid- and long-wavelengths relative to trichromats. It is unclear if dichromacy is advantageous for prey species given predation risks from cryptic species like tigers [13,14,15,16,17]. Understanding the advantages and disadvantages of an animal’s visual system requires knowledge of how specializations have evolved to meet the ecological needs of the animal [7].

Cervidae (Table 1) is a globally occurring family of ungulates that range in size from the <10 kg northern pudu (*Pudu mephitophiles*) to the >750 kg moose (*Alces alces*) [18]. Many human cultures have a long and rich history with cervids and consider them to be an aesthetic symbol of nature or a highly sought after game species [19,20]. Cervids also serve as important ecological drivers within their ecosystems and have both direct and indirect effects on the plant and animal communities within the habitats they occupy [21]. Some examples of these effects include seed [22] and parasite dispersal [23], plant regeneration [24,25], and prey for important apex predators. Cervids rely on visual information for daily behaviors, including predator detection and avoidance, foraging, general movement and navigation, and social interactions. Failure to account for an animal’s perceptual space during research can lead to uninformed conclusions regarding their decision making and behaviors [2,26]. For example, a cervid’s selection of a modest-quality food patch might appear maladaptive given the researchers knowledge of a high-quality food patch nearby. However, the cervid’s selection could represent the best-quality food patch when considering the information from their perceptual space, such as an increased risk of predation at the higher-quality food patch due to dense cover that limits sensory information and, consequently, impedes predator detection. Vision also plays an important role in human–cervid interactions and many approaches to decreasing human–cervid conflicts (e.g., vehicle collisions and crop damage) target vision [27,28,29]. Thus, knowledge of cervid visual ecology provides insights for interpreting behaviors and the development of effective conservation and management strategies. The present review summarizes the literature related to the visual sensory system of cervids in relation to their ecology and highlights knowledge gaps and future research directions to advance our understanding of cervid visual ecology.

## 2. Predator Detection and Avoidance

Common predators of cervids include wolves (*Canis* spp.), coyotes (*Canis latrans*), bears (*Ursus* spp.), big cats (*Panthera* spp.), lynx (*Lynx* spp.), and humans (*Homo sapiens*) [19,76]. Cervids possess many visual characteristics typical of herbivore prey species, including laterally positioned eyes, horizontally elongated pupils [30], dichromatic vision [28,48,49,68], a high-density zone of retinal cells or visual streak [31,34,57], and a reflective tapetum lucidum [57,69,77]. The general visual requirements for the detection and avoidance of terrestrial predators include near panoramic detection of motion and sufficient visual ability in a frontward direction to flee from predators [30]. The lateral placement of cervid eyes provides a wide field of view and minimizes the width of their blind zones for enhanced predator detection, while horizontally elongated pupils and a corresponding visual streak help to facilitate a panoramic field of view while increasing depth of field and minimizing the blur of horizontal contours [30]. Additionally, the spatial association of the horizontal pupil, visual streak, and tapetum allows the eye to capture light along the ground while reducing the capture of incident light from overhead, resulting in an enhanced image quality for features along the ground both at the center and edge of the visual field [30,57,78]. These visual advantages are lost without a mechanism to maintain the horizontal orientation of the eye with the ground. Compensatory cyclovergence, or the simultaneous torsional movement of both eyes, serves as this orientation mechanism [30]. During changes of head pitch, such as the downward movement to graze, this adaptive mechanism maintains the horizontal orientation of the eye with the ground and its functional advantages. 

Beyond their visual streak, other areas of high retinal cell density likely contribute significantly to predator detection and avoidance in many cervid species. For example, frontward locomotion during predator avoidance might be assisted by a dorsotemporal extension of the M-cone visual streak and tapetum in medium-sized (e.g., white-tailed deer, *Odocoileus virginianus* [57]) and large-sized cervids (e.g., moose [31]). The ability of animals to detect predators and flee using visually guided locomotion depends on the spatial and temporal resolution of their visual sensory system [7]. For small-sized cervids (Table 1) such as the southern pudu (*Pudu puda*), the horizontal visual streak of their M-cone photoreceptors likely provides sufficient visual resolution for nearby ground objects [31]. However, medium- and large-sized cervid species (Table 1) might not have sufficient visual resolution for nearby ground objects due to an increased ground-to-eye distance [31]. In both the domestic sheep (*Ovis aries*) and the Nubian ibex (*Capra nubiana*), the dorsotemporal extension of their M-cone topography corresponds with an increase in ganglion cell density at the temporal region of the visual streak and the dorsotemporal region [78,79]. Increased ganglion cell and M-cone density near the temporal and dorsotemporal regions of mid- and large-size cervids might improve the visual resolution in the frontal and lower visual fields. Across Cervidae, the presence or absence of a dorsotemporal extension likely corresponds with species size [31,34]. The total degree of dorsotemporal extension for high-density cell zones of M-cone photoreceptors and ganglion cells likely falls along a continuum based on size, with the characteristic absent in the smallest species (e.g., northern pudu), minimally present in small–medium-sized species (e.g., Reeves’ muntjac, *Muntiacus reevesi*), and well developed in medium–large-sized species (e.g., red deer, *Cervus elaphus*). However, habitat, predation risk, and other factors might also influence the distribution of high retinal cell densities within Cervidae. For example, roe deer (*Capreolus capreolus*) possess an S-cone-enriched ventral retina, a characteristic that is markedly distinct from the S-cone-enriched temporal region of red deer and many other artiodactyls [31,34]. The ventral-oriented topography of S-cone photoreceptors in roe deer likely provides improved chromatic discrimination for overhead cues or signals and plays a crucial role in shaping predator–prey interactions with lynx. Similarly, we anticipate that other cervid species facing comparable predation risks from arboreal hunters might also exhibit a ventral bias in their distribution of S-cones rather than a temporal bias.

Camouflage effectively reduces the salience, or detectability, of an object against its background [80,81]. Generally, dichromats possess a keen ability to break camouflage. Both human and non-human primates with dichromatic vision outperform human and non-human primates with trichromatic vision in searching for camouflaged targets based on texture [13,14,82]. Additionally, in non-human primate species with both dichromatic and trichromatic individuals, dichromats consume more camouflaged prey than trichromats [15,83]. The hypothesized ability of dichromats to break camouflage may be related to their perception of less chromatic noise in their environments [14], especially at low light levels [17]. As dichromats, both cervids and many of their predators are visually adept at detecting and recognizing multiple forms of camouflage. The visual salience of camouflage between cervids and their predators is an ever-evolving interplay as a result of an evolutionary arms race between predator and prey [55,84]. The high salience of a leopard’s pelage for some cervids (e.g., sambar *Cervus unicolor* and chital *Axis axis*) is a result of ongoing selection pressure [85], while the decay of spotted camouflage recognition by black-tailed deer (*Odocoileus hemionus columbianus*) likely results from relaxed selection pressure due to the loss of spotted predators in the last 600,000 years [55]. A counter example exists in Père David’s deer (*Elaphurus davidianus*), which have been isolated from their ancestral predator, tigers, for more than 1200 years, but still respond to the visual and auditory cues of tigers [53]. Furthermore, behavioral responses to forgotten predators might take as little as a single generation of cervids to be restored [86]. Understanding the visual salience of the various forms of camouflage utilized by cervid predators requires a better understanding of cervid sensory ecology, as well as an in-depth understanding of current and past selection pressures.

Multiple aspects of a cervid’s environment have the potential to influence their ability to detect and avoid predators, including light environments. The habitats of cervids exhibit a range of light environments based on vegetative geometry, weather, and time of day [87,88], and light environments likely represent an underappreciated driver of cervid behaviors and decision making [66]. Endler (1993) identified five major diurnal light environments: forest shade, rich in middle wavelengths (yellow-green); woodland shade, rich in short wavelengths, including ultraviolet (blue-gray); small gaps, rich in long wavelengths (reddish); open/cloudy, rich in most wavelengths (“white”); and early/late, deficient in middle wavelengths (purplish). Nocturnal light environments are dominated by middle wavelengths with spectral effects of lunar altitude, lunar phase, and canopy openness, resulting in relative changes in wavelength enrichment [88]. Relative changes in nocturnal light environments influence prey use of habitats, likely as a function of spatiotemporal variations in predation risks [89]. For example, Bawean deer (*Axis kuhlii*) tend to be more active during bright nocturnal periods than dark ones [32], while red brocket (*Mazama americana*) tend to be more active during darker nocturnal periods than bright ones [54]. Variation in light environments affects color perception and contrast sensitivity, which can interact with plant and animal color patterns to make them more or less conspicuous [87,90]. Species-, behavior-, and habitat-specific risk factors likely influence whether and when a cervid is active in conspicuous environments.

Predator pelage that exhibits low near-ultraviolet reflectance may be more conspicuous in ultraviolet-enriched light environments such as a snow-covered woodlands for prey with short-wavelength photosensitivity [91]. Caribou/Reindeer (*Rangifer tarandus*) and white-tailed deer are sensitive to short wavelengths extending into ultraviolet [68,92], and the ocular media of the southern pudu transmits ultraviolet wavelengths [67]. Most cervids are likely sensitive to ultraviolet and near-ultraviolet wavelengths based on ancestral similarities in their ocular media and a lack of ultraviolet-blocking lenses. In caribou/reindeer, ultraviolet sensitivity is rod-mediated at low light levels and is probably S-cone-mediated at high light levels [68]. Despite the lack of specific ultraviolet-sensitive cones in cervids, this spectrum of light is likely important for their ecology, as evidenced by ocular media that selectively increase short-wavelength reflections and facilitate ultraviolet sensitivity in caribou/reindeer [69]. 

Light environments also have dynamic components, which might influence the detection and avoidance of predators by cervids. Dynamic illumination (e.g., dappled forest light) is a common phenomenon in nature that results in increased visual complexity and noise within a light environment [10,81,93]. Many cervid young are born with a cryptic camouflage coloration composed of white spots which mimic dappled light and help to break up the neonate’s outline. Dappled light increases the fixation time of predators and masks the movement of prey [93]. Predator detection of dappled neonates under dynamic illumination might be poor relative to environments with less visual noise. Additionally, mobile prey like cervids may take advantage of these visually noisy and dynamic environments for predator avoidance. Alternatively, dynamic environments may benefit predators by masking their motion. Mule deer avoid forest groves during windy conditions and increased sensory noise, likely because of increased predation risk [56]. Motion limits the efficacy of predator camouflage [94,95], but higher amounts of dynamic visual noise may mask predator motion and maintain the concealment properties of camouflage more effectively. Our inference on this topic is currently limited due to a paucity of research investigating the effects of dynamic illumination on predator–prey interactions for cervids, though the spotted pelage of multiple species of cervid young [96] lends support for its ecological importance and consideration.

The temporal resolution, or ability to track rapid changes in a scene [6,97,98,99], of cervid vision likely plays a key role in the detection and avoidance of predators. The acquisition of visual information at a high temporal resolution is energetically demanding and the temporal requirements of a species’ visual system are shaped by its ecological needs and ability to respond to stimuli [6,100]. As prey species, cervids would benefit from a high temporal resolution to detect and avoid predators. Based on electroretinograms from white-tailed deer (Newman, BA, unpublished data), cervids likely possess a temporal resolution among the highest recorded in non-cervid mammals [6]. Critical flicker fusion, or the frequency at which a flickering light can be perceived as continuous, is often used as a measure of temporal resolution because it can be related to an animal’s ability to rapidly negotiate complex habitats, evade predators, or identify and capture swift prey [6,101,102]. However, critical flicker fusion experiments consisting of high-contrast and luminance stimuli differ significantly from real-world stimuli, which vary in color, size and pattern, luminance, and temporal frequency. Studies of other mammalian visual systems have shown clear differences in temporal resolution at different light levels [103,104] and ages [105]. The temporal resolution of cervid vision across all temporal frequencies is of relevant research interest given that cervids encounter slow-moving obstacles and predators. For example, while a cougar’s (*Puma concolor*) last moments of prey capture are undertaken at a rapid pace, the period prior to ambush involves a slow and methodical stalk of their prey [106]. Additionally, knowledge of the functioning of temporal vision at multiple light levels and age classes is important, since predation risk interacts with these factors. Does a potential degradation in temporal resolution as a cervid ages decrease its ability to detect a slow-moving predator? Or would a lower temporal resolution in young and old cervids limit their reactionary abilities in a predator attack, since an animal’s cognitive processing speed and perception of time might be influenced by the temporal resolution of its visual system [100,107]? To better understand how real-world stimuli are perceived by cervids and the associated risks of this perception, we need to understand the temporal resolution of cervid vision across a wide selection of temporal frequencies and contrasts, as well as a diversity of body sizes, age classes, and diel activity patterns.

## 3. Foraging

Foraging environments are complex systems with spatially and temporally variable distributions of food items that also vary in palatability and nutritional quality. Foragers must effectively locate their preferred food items to maximize their nutritional intake while minimizing energetic search costs and predation risk [108,109]. Herbivores, including cervids, rely on sensory cues (e.g., vision and olfaction) to locate forage patches [38,110], as well as detect the palatability and nutritional quality of food items [111,112]. Multiple aspects of cervid visual systems, including spectral sensitivity and spatial resolution, likely determine the ability of an individual to detect and identify forage items. The extent to which cervid species rely on specific visual systems likely varies because Cervidae exhibits a broad array of selective herbivory between and within species, including fruits, nuts, grasses, browse, forbs, lichens, and fungi across a variety of different habitats. 

Numerous factors, including diet and the diel activity patterns of a species, influence the spatial resolution required to meet their ecological needs [113]. Visual cues of shape and height are often used by mammalian herbivores to identify forage items [112], and might also be used by cervids. Crepuscular or nocturnal species would be predicted to exhibit a relatively low spatial resolution, given that adaptations that enhance visual sensitivity in low light are often incompatible with enhanced contrast sensitivity [113]. Reported measures of visual acuity (i.e., contrast sensitivity measured at the highest spatial frequency) from the crepuscular white-tailed deer [58] and red deer [42] of <6 cycles/degree support this assumption. However, measures of visual acuity alone do not provide a complete frame of reference for spatial resolution and might understate or lead to misinterpretations of the role of detail or contrast discrimination in an animal’s ecology [114]. Contrast sensitivity functions assess spatial resolution over a wide range of spatial frequencies and contrasts, unlike acuity, and provide additional information about the functioning of an animal’s spatial resolution [115]. An understanding of the contrast sensitivity and acuity of cervids is required to assess the role of spatial resolution in cervid visual ecology and foraging behaviors. The spectral qualities of cervid vision are better understood, though still limited, and we can make other inferences regarding cervid use of spectral information for foraging decisions at coarse and fine scales. 

The availability of information at coarse foraging scales depends on the vegetative and light characteristics of an animal’s environment. For example, homogenous environments may provide less visual information for foragers than heterogeneous environments [110], and greater visual obstruction in dense habitats limits the amount of visually available information at coarse scales [1]. Other ungulates use visual cues to forage efficiently in fixed and variable environments [110,116]. Coarse-scale foraging decisions (e.g., patch selection) in cervids might be informed by visual cues associated with high- or low-quality foraging opportunities, such as color or the amount of visual obstruction. For example, roe deer may use the contrasting colors of distant pines or willows to locate mixed forage patches [38]. Cervid species may rely on visual cues from the environment for coarse-scale foraging decisions to varying degrees depending on environmental characteristics, species-specific foraging strategies, and the limitations of their visual systems.

Fine-scale foraging decisions are likely dependent upon a complex interaction of sensory information, including aspects of cervid vision. Cervids may use their ability to distinguish color at certain wavelengths to identify preferred food items. Surface vegetation, including lichen, appear in high achromatic contrast against snow in ultraviolet-only images [72,117]. Given the sensitivity of caribou/reindeer to ultraviolet wavelengths [68], they likely experience improved forage discrimination in their ultraviolet-dominated light environments [72,117]. Many fungi and plants emit or reflect ultraviolet cues [118,119], and ultraviolet sensitivity might play a role in fine-scale forage discrimination for cervids. In addition to ultraviolet wavelengths, other short- and medium-wavelength stimuli likely play a role in fine-scale forage discrimination for cervids. During fine-scale foraging experiments, cattle (*Bos taurus*) used visual color cues to differentiate green forage from dead forage [120,121], while sheep were found to be capable of distinguishing similar chromatic hues (i.e., green and yellow) based on brightness [122]. Similarly, cervids might use visual cues of hue and brightness to distinguish high- and low-nutrient forage, as well as potential toxins at fine scales. Researchers have suggested that the color vision of fallow deer (*Dama dama*) might help them forage more effectively [49]. The presence of color vision in cervids is likely at least partially founded from the ecological need to efficiently identify quality forage. However, the investigation of visual physiology, as it relates to the acquisition of foraging information in Cervidae, has received minimal research attention. 

Based on electrophysiological measurements and behavioral trials, cervids possess S-cone, M-cone, and rod photoreceptors with species-specific variations in peak sensitivities [47,48,59,68,92]. The spectral tuning of vertebrate visual pigments is generally considered to be adaptive [123,124] and species-specific variations in peak sensitivities within Cervidae might be linked to species-specific foraging strategies. The spectral tuning of dichromats in forests is independent of spectral illuminants [125], and differences in peak sensitivities in cervids, particularly forest-dwelling species, could relate to the spectral properties of their forage items and periods of activity. Cervids fall within a frugivore–browser–grazer continuum of foraging strategies [126]. Currently, no information exists on the species-specific spectral sensitivity of Cervidae’s strictly frugivorous genera (e.g., *Muntiacus* spp.), and exploring the role spectral tuning plays in the acquisition of visual information is of interest for these species. Evidence of spectral tuning in ocular media other than photopigments can be found within Cervidae. Caribou/Reindeer experience a seasonal shift in tapetum lucidum color, which is spectrally tuned for Arctic-twilight conditions [69,70,127]. While not yet experimentally evaluated, the spectral tuning of caribou/reindeer tapetum reflectance likely assists in greater photon capture during low-light conditions and the detection of forage. More research is needed to understand the prevalence of the spectral tuning of ocular media within Cervidae to understand its role in the efficient acquisition of visual information during foraging.

## 4. Movement and Navigation

Movement and the ability to navigate efficiently represent important aspects of an animal’s ecology. Mobile species gain adaptive advantages by moving through their environments in such a way as to optimize their chances of survival and reproduction [128]. When, where, and how an animal selects to move through their environment is ultimately influenced by their available spatial information [26,128]. Cervids, like multiple non-cervid mammals, rely on idiothetic (internal) and allothetic (external) information to determine their spatial position. Idiothetic cues relate to an animal’s self-motion-based information like optic flow (i.e., global visual changes during motion) and allow an animal to assess how its own movement has affected its spatial position [128], while allothetic cues incorporate space-defining information in the environment such as beacons, landmarks, and environmental boundaries. Allothetic information related to distal visual cues and environmental geometry is particularly important in establishing orientation and location, but idiothetic information plays a vital role in tracking movement and in stabilizing and extending the representation of location into open spaces [128]. Reliance upon a single information system over another might depend upon an animal’s rate of movement, the size and structure of their environment, and prior experience within that environment [129]. For example, high travel speeds create a velocity blur that constrains the distance and width of effective visual searches to obtain allothetic information. More generally, idiothetic and allothetic information likely works in conjunction in most situations to limit spatial error [128,129].

The maternal care strategy of ungulates for avoiding predation can be classified into two categories: followers and hiders [96]. The vast majority of cervids exhibit a hider strategy, wherein young lie concealed away from their mother while she forages [96]. Hiders use this strategy sometimes for multiple weeks before eventually joining their mother during foraging activities. Mothers with hidden young must rely on their ability to use idiothetic and allothetic information to navigate their environments and locate the general location of their hidden young after foraging bouts. As previously outlined, vision provides a better spatial accuracy than auditory or olfactory information, though its detection field is generally smaller [2,26], and the ability to navigate using visual–allothetic cues likely contributes to the reliable ability of mothers to find their hidden young. More generally, cervids are known for their high-site fidelity within and across seasons, and the ability to maintain these fine-scale spatial ranges likely depends on visual spatial information and memory [130,131].

Migration is a fundamental behavior of many ungulate species, including some cervids. Migration often allows species to sustain larger populations than their resident counterparts and links ecosystem processes across large spatial scales [132,133]. Most importantly, migration provides access to critical resources that can differ by location and season. For example, migratory species are most likely to occur in regions with resource waves across the landscape [132,133,134], which makes these long-distance coordinated movements more advantageous despite their spatial complexity. Both mule deer and red deer jump the “green wave” during migration [135,136], while also being able to flexibly modify their habitat selection to track green-up on smaller scales. However, spatial memory likely exerts the strongest influence on migration to seasonal ranges [137,138]. The hippocampus, a widely studied mammalian brain structure, plays an integral role in memory formation. Interestingly, the hippocampus of many ungulates is morphologically distinct in a region associated with spatial learning [139]. The elongation of this specialized brain structure suggests that ungulates, including cervids, might possess an enhanced ability to integrate topographical features and other objects for spatial navigation [139]. Additionally, the low spatial resolution of cervid vision [42,58] coupled with their wide field of view might provide robust orientation abilities for cervids. Visual information for homing often resides in lower spatial frequencies and low spatial resolution in many species likely represents a beneficial adaptation for navigation, and not necessarily a compromise of performance [140]. Experimental and observational exploration of view-based navigation strategies for cervids is necessary to fully understand how visual information is used to navigate short and long distances.

## 5. Social Interactions

Many cervids are social animals that need to process social information efficiently from various sensory systems. The importance of vision for conspecific and kin recognition has received limited attention for cervids. Other ungulates are able to identify conspecifics and kin based on visual information [141,142]. Domestic sheep are able to discriminate between images of conspecific faces across ages and in different orientations (i.e., frontal vs. profile views) [141]. Ewes use visual cues for aid in identifying their young from alien young [143]. The spatial resolution of domestic sheep measures up to 14 cycles per degree [144], however, a high spatial resolution is not a defining feature for facial and kin recognition. Domestic cattle have a spatial resolution comparable to or below that measured in cervids, yet cattle have efficient individual recognition based on visual information [142]. More research is needed to understand the individual recognition abilities of cervids and the role that this ability plays in social interactions such as parent–offspring identification or non-kin recognition. 

Certain color variations in specific areas of cervid pelage have visual signaling functions. For instance, white regions on the hind pelage of many cervids have a visual signaling function likely aimed at conspecifics or, potentially, predators [145]. Cervids that live in larger social groups are more likely to have conspicuous hindquarters [96,146]. The specific shape and size of hind pelage coloration varies considerably across Cervidae, and, in some instances, even within a species or season, ranging from large encompassing patches to more discrete coloration that can be easily hidden by the tail [76,147]. For example, white-tailed deer can selectively expose the white underside of their tails, known as tail flagging. This behavior is performed by all ages and both sexes, and the frequency of flagging does not appear to differ between doe groups composed of female relatives and buck groups composed of nonrelatives. Therefore, tail flagging in cervids likely serves as a signal that helps to maintain group cohesion for antipredator benefits [51,60]. Tail flagging may also inform an approaching predator that it has been detected [148]. Additionally, white- and black-tailed deer, which display white rumps and tails during escape but hide them when stationary, might be using flash behavior to confuse the predator into looking for the wrong object and thereby avoid detection [145]. Only conspicuous flash displays effectively reduce predation risk [149], so having a pelage capable of reflecting all wavelengths of light (i.e., white) which can be hidden and displayed when necessary would serve as an effective distraction tactic for predators, as well as a threat signal for conspecifics. Other contrasting coloration patterns, such as the overt darkening of nuptial pelage found in many cervids [96,147] or the more subtle labial spots present in some cervids [150], are likely involved in intraspecific communication. While the precise role of contrasting pelage coloration in both intraspecific and interspecific communication remains uncertain, it merits thoughtful consideration, including the potential for the species-specific coding of these visual signals and others.

Highly developed facial musculature enables cervids to display a variety of facial expressions using the mouth and ears, while control of their body positioning and the local erection of hair provides an additional breadth of visual communication potential. Cervids are known to use the posture of the head and facial expression to provide visual signals to other members of the herd or to territorial or sexual rivals. For example, wapiti (*Cervus canadensis*) elevate their nose and the upper lip above the canines is sharply raised during some threat displays, chital are thought to use differences in head height to communicate play and threat signals [76], and caribou/reindeer use repeated head posturing to attract their young [151]. Expressive dominance displays by male and female cervids include body posturing, piloerection, ear drop, and flaring of the preorbital gland and nostrils. Female moose often display a head low threat with local piloerection along the neck and shoulders, which increases their perceived body size during the defense and protection of their young [151]. Male cervids use stiff, erect, or stretched body postures with either head up or down threats, depending on the signal intent (e.g., a weapon threat vs. courtship display). Ear drop and flaring of the preorbital gland and nostrils can also signal serious agonistic or sexual intent in cervids [74,75]. For example, female rusa deer (*Rusa timorensis*) flare their preorbital glands during agonistic behaviors with other females [75]. In contrast, preorbital gland opening in juvenile red deer is associated with milk solicitation and is thought to be a visual indicator of satiety [43]. Despite their noted relevance for visual communication [152], the many glands of cervids have received limited research attention to understand their role in visual communication more specifically.

Multiple cervid species are known for their distinctive antlers that function as important social organs. Visual displays of antlers by males often precede social interactions including combat (i.e., fighting or sparring) and reproductive pursuits [76,147,151,153]. For example, the lateral presentation of antlers can visually signal submission or an appeasement gesture to de-escalate a potentially agonistic situation with a conspecific [153,154], while the lateral presentation of the body and antlers in a parallel walk between contesting conspecifics is often associated with agonistic interactions [52,151,153,155,156]. Interestingly, though the exact information conveyed in a parallel walk remains unclear [52,157], it is unlikely the information would be as effectively communicated without motion [10]. Given the low spatial resolution of cervid vision, motion during visual displays might amplify and enhance these signals and play a crucial role in signal acquisition by conspecifics. Some cervid species, including caribou/reindeer and fallow deer, perform showy displays during the courtship of females that involve repetitive motions of the head and antlers [76,147]. Courtship displays and their specific movements, such as a swaying presentation of antlers, might represent an important component of female signal acquisition and potential mate evaluation in cervids. Because antlers provide an honest signal of genetic quality [36,158], females might prefer males with larger and more complex antlers because they are a fitness correlate. For example, relative antler size and complexity are associated with measures of male fertility in red deer [158] and the probability of becoming a harem holder [159]. Additionally, female white-tailed deer prefer larger-antlered males to smaller-antlered males when intrasexual competition is controlled, making antlers both a weapon and an ornament [61]. It may be advantageous for females to choose mates with larger and more complex antlers if they produce “sexy sons” with similar traits, who, in turn, also have greater reproductive success [160]. However, the signaling function of antlers might be suppressed in the presence of male intrasexual competition or in lekking cervids. For example, captive-female fallow deer did not show a preference for antlers and instead selected males based on the presence of other females [161], which could be a result of differences in female discrimination between males based on alternative characteristics or their mating system. An additional investigation of female preference is necessary to understand the relative importance of male traits in sexual selection across Cervidae and the role of visual cues in this choice.

Cervid species also communicate using visual cues, or signposts, that accompany and draw attention to olfactory cues [39,50,62]. For example, roe bucks, when defending mating territory, clear the surrounding vegetation near their scent mark, which improves signal localization relative to airborne olfactory cues alone [39]. Unsurprisingly, more conspicuous visual markings receive greater conspecific attention than less conspicuous marks [50]. The use and importance of signposts vary throughout the year and across cervid species, and their design similarly differs among species and likely depends on available habitat, social structure, and function [76]. A ubiquitous characteristic of signposts is urine marking. Given the ultraviolet sensitivity of cervids [48,67,68], it is possible that urine marking at signposts and on individuals might serve as an additional visual indicator of olfactory signal presence. The urine of various rodents fluoresces in ultraviolet [162] and species like the desert iguana (*Dipsosaurus dorsalis*) use ultraviolet visual markers for locating low-volatility pheromones in the environment [163]. Further research to understand the degree of ultraviolet sensitivity present in cervids and the spectral properties of their urine would help to clarify if urine staining and marking in Cervidae also provides a visual signal for detection and localization.

## 6. Human–Cervid Interactions

Globally, we are in a rapid state of change with expanding human communities and shifting climate patterns. Many cervid species find human communities to be a suitable habitat and, in some cases, seek out these habitats, increasing human–cervid interactions. Worldwide, expanding cervid populations impact ecosystem dynamics [21], browse on economically important crops [164], increase risks of vehicle collisions [165], and contribute to a risk of disease spread among wildlife, humans, and livestock. For example, sika deer (*Cervus nippon*) contribute to more than 50 million dollars in agricultural damage every year in Japan [164], and, in the United States, millions of cervid–vehicle collisions occur annually, posing a threat to wildlife and human health, as well as causing economic damage for drivers [165,166]. Despite incurring substantial economic damages and representing a considerable threat to human health, our ecological understanding and development of effective deterrents is minimal. 

Exclusion methods like fencing can be effective at limiting human–cervid conflict and cervid damage. Behavioral responses to fences vary by cervid species [167,168], and an individual’s visual perception of a barrier or fence can influence penetration [65,169]. But effective fences can be costly and difficult to maintain [170], as well as negatively impact wildlife movement and connectivity. Therefore, other methods for limiting economic damages and threats to human health from human–cervid interactions are often desirable. Many of these deterrents target the various sensory systems of cervids, like vision. Wildlife warning reflectors are marketed as effective visual deterrents for reducing cervid–vehicle collisions and consist of reflective mirrors that redirect light from oncoming vehicles. However, multiple investigations of reflector effectiveness in reducing cervid–vehicle collisions have produced variable results, with many concluding that the deterrents were ineffective or not reliably effective [171,172,173]. An insufficient understanding of cervid behavior and visual perception of vehicles and roadways constrains our ability to develop effective deterrents using visual signals and cues. Currently, the mechanism that causes the well-known “deer in the headlights” phenomenon is still unclear. Does the sensory information from headlights overwhelm cervids, does the stimulus produce an insufficient looming cue to indicate danger, or could the stimulus produce contradictory danger cues? Based on the low spatial resolution of cervid vision and the diffraction of light, oncoming headlights, from a cervid’s perspective, might appear to laterally split upon approach. White-tailed deer exhibit differences in behavior (i.e., flee or freeze) based on the total frontal area of a vehicle that is illuminated [27]. However, this behavioral shift is still being explored and, at this time, has not been evaluated in other cervid species. In general, a better understanding of cervid visual ecology is necessary to develop effective technologies for deterring cervids based on visual signals and cues.

Finally, we need to understand how the altered landscapes associated with human expansion and growth affect cervids. Many of our urban and suburban environments are filled with light, particularly short-wavelength light. How do these human-altered light environments interact with and influence cervids? As a result, what adaptations might be lost or gained over time in these new environments? Evaluating the impacts of human expansion on cervids is difficult, because not all effects are overt, and failure to account for differences in visual perception can lead to overlooking potentially problematic alterations. For example, high-voltage power lines can produce corona discharge, a light-emitting phenomenon that peaks in UV and can produce an illusion of motion. Corona discharges are mostly outside of the visual perceptual range of humans, but well within the spectral sensitivity of caribou/reindeer. Caribou/Reindeer tend to avoid power lines, and this might be a result of their ability to see corona discharges and a perceived risk [71,73]. Thus, consideration of cervid visual ecology is necessary to accurately determine and predict the effects of human-altered landscapes on cervids.

## 7. Conclusions and Future Directions

Cervidae consists of many unique species of varying ecological niches. At the current time, only a select few cervid species have received in-depth research attention regarding the basic physiological aspects of their visual systems. Given the wide range of sizes, social structures, and habitats within Cervidae, more basic research is needed on multiple understudied species around the world. For example, representative retinal cell topographies, photoreceptor sensitivity, and basic measures of spatial and temporal resolution are needed from multiple species, representing the diversity of habitats, activity patterns, and sizes found within Cervidae. We can then begin to ask more complex questions regarding cervid visual ecology, such as what aspects of cervid visual perception influence their behaviors and decisions during foraging, what factors influence a cervid’s ability to detect a camouflaged predator, or how cervids use their visual systems for navigation. As our knowledge of their current visual adaptations and specializations grows, so will our understanding of how they evolved, our ability to predict the realities of global change on these specializations, and our ability to effectively conserve and manage cervids, including human–cervid interactions.

## Figures and Tables

**Table 1:** Summary of cervid species, their geographic distribution, estimated shoulder height (SH; cm), body mass (BM; kg) [18], conservation status based on the IUCN red list categories (DD, data deficient; LC, least concern; NT, near threatened; VU, vulnerable; EN, endangered; and CR, critically endangered), and research investigations of physiology, signals, and behavior related to visual systems.

Common Name	Scientific Name	Distribution	SH	BM	Status	Vision Research
Moose	*Alces alces*	North America, Europe, Asia	185–210	280–600	LC	Physiology [30,31]
Chital	*Axis axis*	south Asia	70–95	45–85	LC	
Calamian deer	*Axis calamianensis*	Calamian Islands	60–75	35–50	EN	
Bawean deer	*Axis kuhlii*	Bawean Island	60–75	40–60	CR	Behavior [32]
Indian hog deer	*Axis porcinus*	South and southeast Asia	55–75	30–55	EN	
Marsh deer	*Blastocerus dichotomus*	South America	100–130	70–130	VU	Physiology [33]
European roe deer	*Capreolus capreolus*	Europe, west Asia	65–84	17–30	LC	Physiology [31,34,35]; Signals [36,37,38]; Behavior [29,39]
Siberian roe deer	*Capreolus pygargus*	Asia	82–94	32–50	LC	Behavior [40]
White-lipped deer	*Cervus albirostris*	China	110–130	90–220	VU	
Wapiti	*Cervus canadensis*	North America, Asia	130–165	150–400	LC	Signals [41]
Red deer	*Cervus elaphus*	Europe, west Asia	95–130	75–220	LC	Physiology [31,34,35,42]; Signals [43,44]; Behavior [45]
Central Asian red deer	*Cervus hanglu*	Central Asia	110–145	110–240	LC	
Sika deer	*Cervus nippon*	East Asia	60–115	20–140	LC	Physiology [46,47]
European fallow deer	*Dama dama*	Europe	70–95	35–80	LC	Physiology [48,49]; Signals [50,51,52]
Persian fallow deer	*Dama mesopotamica*	Iran, Israel	90–110	70–140	EN	
Tufted deer	*Elaphodus cephalophus*	China	50–70	17–30	NT	
Père David’s deer	*Elaphurus davidianus*	China	110–140	140–220	EW	Signals [53]
Taruca	*Hippocamelus antisensis*	South America	70–80	45–60	VU	
Patagonian huemul	*Hippocamelus bisulcus*	Argentina, Chile	80–90	60–75	EN	
Water deer	*Hydropotes inermis*	China, Korean Peninsula	50–55	11–15	VU	
Red brocket	*Mazama americana*	South America	60–80	30–35	DD	Physiology [33]; Behavior [54]
Small red brocket	*Mazama bororo*	Brazil	50–60	25	VU	Physiology [33]
Mérida brocket	*Mazama bricenii*	South America	45–50	8–13	VU	
Peruvian dwarf brocket	*Mazama chunyi*	Bolivia, Peru	38	11	VU	
Gray brocket	*Mazama gouazoubira*	South America	50–65	11–25	LC	Physiology [33]
Brazilian dwarf brocket	*Mazama nana*	South America	45–50	14–16	VU	Physiology [33]
Amazonian brown brocket	*Mazama nemorivaga*	South America	50	14–16	LC	Physiology [33]
Dwarf red brocket	*Mazama rufina*	Colombia, Ecuador, Peru	45	10–15	VU	
Central American red brocket	*Mazama temama*	Central America, Colombia	60–70	12–32	DD	
Bornean yellow muntjac	*Muntiacus atherodes*	Borneo	65	14–18	NT	
Black muntjac	*Muntiacus crinifrons*	China	55	20–25	VU	
Fea’s muntjac	*Muntiacus feae*	Myanmar, Thailand	50–60	20–22	DD	
Gongshan muntjac	*Muntiacus gongshanensis*	China, Myanmar	55	20–25	DD	
Southern red muntjac	*Muntiacus muntjak*	Southeast Asia	50–70	20–35	LC	
Pu Hoat muntjac	*Muntiacus puhoatensis*	Vietnam	40		DD	
Leaf muntjac	*Muntiacus putaoensis*	India, Myanmar	40	12	DD	
Reeves’ muntjac	*Muntiacus reevesi*	China, Taiwan	45–50	12–15	LC	Physiology [31]
Roosevelts’ muntjac	*Muntiacus rooseveltorum*	Laos	40		DD	
Annamite muntjac	*Muntiacus truongsonensis*	Laos, Vietnam	40	15	DD	
Northern red muntjac	*Muntiacus vaginalis*	South and southeast Asia	50–70	20–28	LC	
Giant muntjac	*Muntiacus vuquangensis*	Vietnam, Laos, Cambodia	65–70	34	CR	
Mule deer	*Odocoileus hemionus*	North America	75–105	35–110	LC	Signals [55]; Behavior [56]
Yucatan brown brocket	*Odocoileus pandora*	Yucatan Peninsula	70	17–21	VU	
White-tailed deer	*Odocoileus virginianus*	North and South America	55–105	25–130	LC	Physiology [28,33,48,57,58,59]; Signals [60,61,62,63]; Behavior [64,65,66]
Pampas deer	*Ozotoceros bezoarticus*	South America	60–70	22–40	NT	Physiology [33]
Northern pudu	*Pudu mephitophiles*	Colombia, Ecuador, Peru	25–38	5–6	DD	
Southern pudu	*Pudu puda*	Argentina, Chile	30–40	9–14	NT	Physiology [31,67]
Caribou/Reindeer	*Rangifer tarandus*	North America, Europe, Asia	70–135	55–170	VU	Physiology [67,68,69,70]; Signals [71,72,73]
Barasingha	*Rucervus duvaucelii*	India, Nepal	115–135	140–200	VU	Signals [74]
Eld’s deer	*Rucervus eldii*	South and southeast Asia	90–130	60–125	EN	
Visayan spotted deer	*Rusa alfredi*	Philippines	65–75	40	EN	
Philippine deer	*Rusa marianna*	Philippines	55–70	40–60	VU	
Javan deer	*Rusa timorensis*	Indonesia	85–110	50–135	VU	Signals [75]
Sambar	*Rusa unicolor*	South and southeast Asia	110–160	130–270	VU	

## Data Availability

Original contributions presented in this review are included in the article, further inquiries can be directed to the corresponding author.
